# On the Localising of Fractures of the Base of the Skull

**Published:** 1895-07-27

**Authors:** Alexander Miles

**Affiliations:** Surgical Tutor, Royal Infirmary, Edinburgh


### Medical Progress and Hospital Clinics.

[*The Editor will be glad to receive offers of co-operation and contributions from members of the profession. All letters should be addressed to* The Editor, The Lodge, Porchester Square, London, W.]

### ON THE LOCALISING OF FRACTURES OF THE BASE OF THE SKULL.

By Alexander Miles, M.D., F.R.C.S.Edin., Surgical Tutor, Royal Infirmary, Edinburgh.

It is often by no means easy in cases of severe injuries to the head to make even an approximate diagnosis as to the exact seat of the lesions in the brain or skull, on account of compression rendering the patient quite unconscious, and obscuring all localising symptoms of irritation or paralysis. While it cannot be said that an accurate diagnosis of the precise position of a basal fracture influences the line of treatment to any great extent, it is nevertheless highly desirable that the surgeon should make up his mind on the point, in view of the bearing it has on the prognosis, both immediate and remote. Excluding the cases in which the fracture of the base of the skull is the result of such direct violence as a stab in the orbit, nose, or pharynx, or of gunshot wounds, and in which there is, therefore, necessarily no difficulty in determining which fossa of the skull is involved, it may be said generally that basal fractures are usually caused by injuries more or less diffused in their nature, and that the seat of impact of the blow is a guide, although not an infallible one, to the fossa fractured. When the injury is caused by a pointed or sharp-edged instrument, the fracture is commonly of the vault, and the base escapes. While it is convenient, in considering the differential diagnosis of fractures involving the separate fossæ of the base, to look at the points of distinction peculiar to each, it is in no way inferred that fractures are strictly limited to one or other clinically. On the contrary, the fissures which radiate from the main break not unfrequently spread into the neighbouring fossæ, and, by complicating the signs and symptoms, add to the difficulty of the diagnosis.

1. **Fractures of Anterior Fossa.**—These are often the result of direct violence from such accidents as the entrance of a fencing foil, or the end of a stick or umbrella, through the orbit, and there is then no difficulty in determining the locality of the lesion. Other causes of such fractures are blows over the face, nose, or forehead, in which cases the force is transmitted to the basal region through other bones, and the violence is said to be indirect. A blow on the nose, forcing back the nasal bones against the ethmoid fractures the latter, and so involves the anterior fossa, the fracture being a compound one. Blows over the frontal bone frequently result in fracture of the orbital plates, in which case the fracture is usually simple. When external evidence of fracture of the anterior fossa exists, it is usually in the form of bleeding from the nose, mouth, or pharynx, followed or accompanied by the escape of cerebro-spinal fluid, and in a few rare instances the escape of small portions of brain matter. The two last-named signs leave little doubt as to the existence of a fracture and its site, but it is obvious that the significance of epistaxis may easily be misinterpreted. When the blood has an intra-cranial source, the torn vessels are usually those of the meninges, much less frequently the large venous sinuses. In many cases the blood does not escape externally, but passes into the tissues of the orbit and parts around. Under such circumstances care must be taken to exclude the possibility of the signs being due to a simple black eye, or to the results of blood gravitating from an injury on the frontal region as so often occurs. The points to note in this connection are (1) the relation of the extravasated blood to the conjunctiva, and the period of its appearance; (2) which eyelid is the seat of ecchymosis, when it occurred, and the direction of its spread. No deduction can safely be drawn from variations in the colour of the ecchymosis in different cases. In the case of an ordinary black eye the effusion into the eyelids comes on very rapidly, and affects chiefly the lower lid. When sub-conjunctival ecchymosis exists at all, it also is an immediate result of the injury, being due to laceration of the vessels of that membrane, and not to gravitation. When black eye is simulated by blood gravitated from the forehead, the discoloration naturally appears first in the upper eyelid, and is only detected a considerable time (probably two or three days) after the accident. Sub-conjunctival effusion is uncommon. When and where the effusion takes place in fractures of the anterior fossa depends on whether the fracture involves (*a*) the orbital plate, or (*b*) the margin of the orbit. The latter lesion is not common, but every now and again occurs, and is accompanied by speedy and well-marked discoloration first of the upper lid, later of the lower, accompanied by effusion under the conjunctiva. When the orbital plate is fractured the blood is at once poured into the tissues of the orbit, causing a forward projection of the eye, a triangular patch of ecchymosis appears at once under the conjunctiva, usually at the outer angle of the eye. After a varying interval of time the lower lid gradually becomes discoloured, and the upper lid may or may not follow suit. Paralysis of one or more of the orbital nerves is not uncommon in fractures of the anterior fossa. Blindness from pressure on the optic nerve; external squint, dilatation of the pupil, and drooping of the upper lid from involvement of the third nerve; or internal squinting from interference with the sixth nerve may result. Cerebral symptoms referable to a distinctly localised brain lesion are very uncertain. When they do supervene it is usually after an interval of at least a few days, and they are due to the results of septic infection and pus formation.

2. **Fractures of the Middle Fossa.**—This is undoubtedly the most common situation of basal fracture. The seat of impact of the blow may be the vertex, the occiput, or the lateral aspects of the head. Not unfrequently falls on the feet or on the buttocks lead to the same lesions. The classic signs of this fracture are bleeding from the ear, accompanied or followed by the escape of cerebro-spinal fluid, with facial paralysis. Deafness on the same side is strongly corroborative, and the escape of brain tissue places the diagnosis beyond doubt. In investigating such injuries there are certain fallacies to be guarded against. (1) The blood seen in the ear may have flowed there from a scalp wound—careful cleansing of the meatus and the use of an ear speculum will settle the point; (2) a rupture of the tympanic membrane, or a fracture of the external auditory meatus, may give rise to an escape of blood from the ear, but it will be in bright arterial jets, ceasing early, in contradistinction to the long-continued oozing of dark-coloured blood which follows on a basal fracture; (3) every now and again bleeding and welling of cerebro-spinal fluid occurs in cases where no basal fracture exists, but as this can only be proved by post-mortem examination, in considering clinical diagnosis the fact must be ignored; (4) in a few cases of fracture of the middle fossa blood has escaped from the mouth by way of the Eustachian tube, the tympanic membrane being intact. The escape of cerebro-spinal fluid, which is often very profuse, continuing for many days, means the opening up of the cavities of the brain membranes, and indicates the greatest care in the use of antiseptic precautions to prevent access of infective organisms. In fractures of this fossa the cerebral symptoms vary with the degree of compression which exists, and of themselves are of comparatively little value as diagnostic guides to the seat of the fracture.

3. **Fractures of Posterior Fossa.**—Patients suffering from this fracture are usually rendered unconscious by serious damage being done to the important parts of the brain which occupy this fossa of the skull, and distinctive features of these fractures are by no means prominent. The injury is usually received on the occiput, or from a fall on the buttocks, in which case the force impinges on the condyles of the occipital bone. An effusion of blood behind the mastoid process, or among the muscles of the back of the neck sometimes appears, but not for some days after the accident. Symptoms referable to special nerves are seldom present, evidences of pressure on the pons and medulla, with marked respiratory difficulty, being the prominent features. When the mastoid process is broken across air may escape into the tissues around, giving rise to emphysematous crackling.

**Percussion Note.**—In all cases of suspected fracture of the skull the percussion note should be tested. Professor Macewen has employed this test for many years, and attaches considerable importance to it as an aid in the diagnosis of intra-cranial lesions. In cases of basal fracture the writer has occasionally detected a difference on the two sides of the head, but as this is frequently to be made out on intact skulls the value of the sign is diminished. Much more valuable, however, is it to find that the patient himself observes a difference in his sensations when opposite sides of the skull are percussed. This the writer has frequently observed in some cases, the patient alleging that the difference is very marked.

